# A road surface image dataset with detailed annotations for driving assistance applications

**DOI:** 10.1016/j.dib.2022.108483

**Published:** 2022-07-23

**Authors:** Tong Zhao, Yintao Wei

**Affiliations:** State Key Laboratory of Automotive Safety and Energy, Tsinghua University, Beijing, China

**Keywords:** Road surface classification, Road unevenness, Tire-road friction, Computer vision, Intelligent vehicles, Autonomous driving

## Abstract

The preview of the road surface states is essential for improving the safety and the ride comfort of autonomous vehicles. The created dataset in this data article consists of 370151 road surface images captured under a wide range of road and weather conditions in China. The original pictures are acquired with a vehicle-mounted camera and then the patches containing only the road surface area are cropped. The friction level, material, and unevenness properties of each road image are annotated in detail. This large-scale dataset is useful for developing vision-based road sensing modules to improve the performance of the driving assistance systems. Also, deep-learning experts can regard this dataset as a comparing benchmark for their algorithms. The dataset is available at [1].

## Specifications Table

**Table:** 

Subject	Computer Vision and Pattern Recognition
Specific subject area	Vision-based Road Surface Classification with Deep Learning for Driving Assistance Purpose
Type of data	RGB Image
How the data were acquired	The original pictures are captured with a USB camera mounted on the bonnet. The camera is controlled by an IPC running a Python script. Then the road area of the original pictures is cropped into patches manually.
Data format	Raw Images(.jpg)
Description of data collection	The pictures are acquired at daylight with a variety of weather conditions. Roads of different materials, service ages, and city regions are covered in the dataset. The vehicle moves with a velocity in the range of 20-80km/h. Five pictures are collected per second. Many corner cases such as garbage on the road, dirt on the lens are also contained.
Data source location	City: BeijingCountry: China
Data accessibility	Repository name: Mendeley/ZenodoData identification number: doi:10.17632/w86hvkrzc5.2Direct URL to data: https://doi.org/10.17632/w86hvkrzc5.2Code identification number: doi:10.5281/zenodo.6812787Direct URL to code: https://doi.org/10.5281/zenodo.6812787

## Value of the Data

• This large-scale dataset lays the basis for identifying road surface conditions with vehicle-mounted cameras and is useful for researchers to develop road sensing and driving assistance systems.
• To the best of the author's knowledge, this is the first dataset that simultaneously annotates the friction level, unevenness, and material properties of the road images. The class definition of each road property is reasonable and specific.
• Further researches correlated with road monitoring and accident prevention can also be conducted based on the whole or part of the dataset [2,3].
• The dataset covers as many available working conditions as possible. The robustness of the algorithm to be developed can be guaranteed on the dataset level.
• For deep learning experts, this dataset can act as a benchmark to compare the performance of different image classification algorithms.

## 1 Data Description

Road conditions sensing with image data is verified to be feasible in providing essential information for vehicle control systems [4,5]. Most existing public datasets contain images collected under limited working conditions, which restrict the robustness of the algorithms in practical applications. This large-scale dataset annotated the friction level, material, and unevenness properties of road images acquired under various conditions.

The friction level property contains six subclasses corresponding to different weather conditions, i.e. dry, wet, water, fresh snow, melted snow, and ice. The road material property consists of asphalt, concrete, mud, and gravel. The road unevenness is divided into smooth, slight unevenness, and severe unevenness according to the amplitude of the road undulation. The subclasses of the three road properties are combined to form the class definition of the dataset. It should be noted that the road material and unevenness are not annotated when the friction levels are fresh snow, melted snow, or ice. Also, the unevenness is not labeled for mud or gravel roads.

**Fig. 1:**
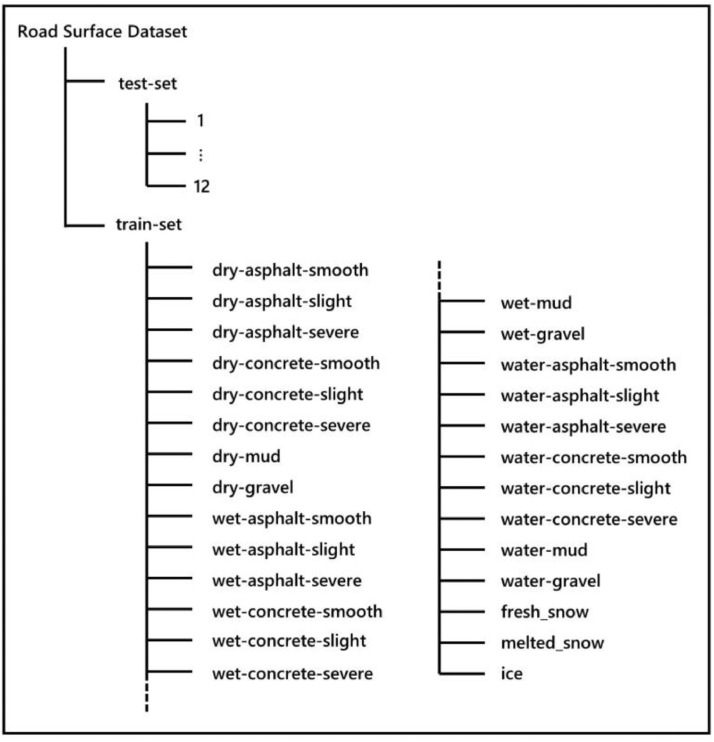
The dataset directory structure.

**Fig. 2:**
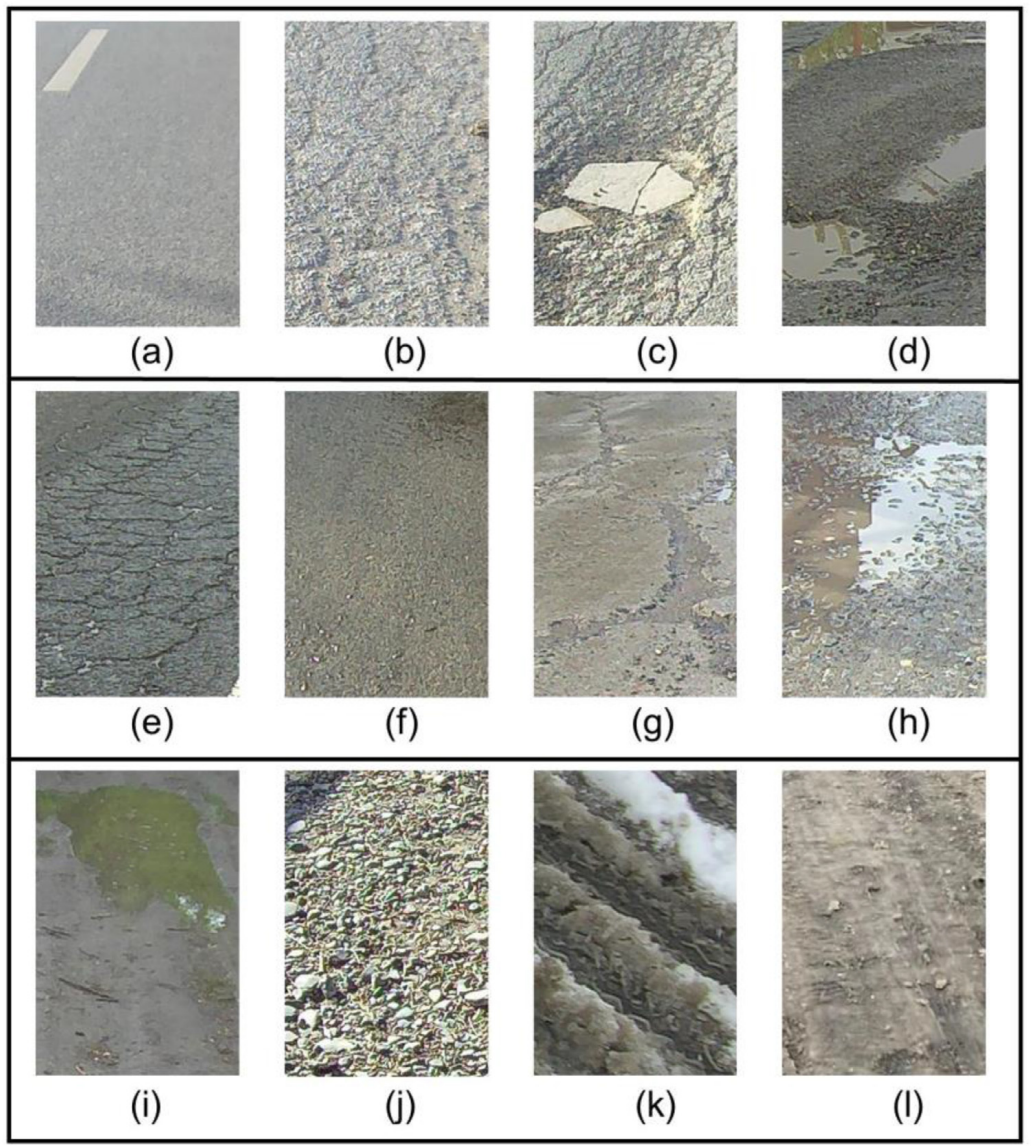
Sample images of part of the classes. (a). dry-asphalt-smooth (b). dry-asphalt-slight (c). dry-asphalt-severe (d). water-asphalt-severe (e) wet-asphalt-slight (f). wet-concrete-smooth (g). wet-concrete-severe (h). water-concrete-slight (i). water-mud (j). dry-gravel (k). melted snow (l). ice.

**Fig. 3:**
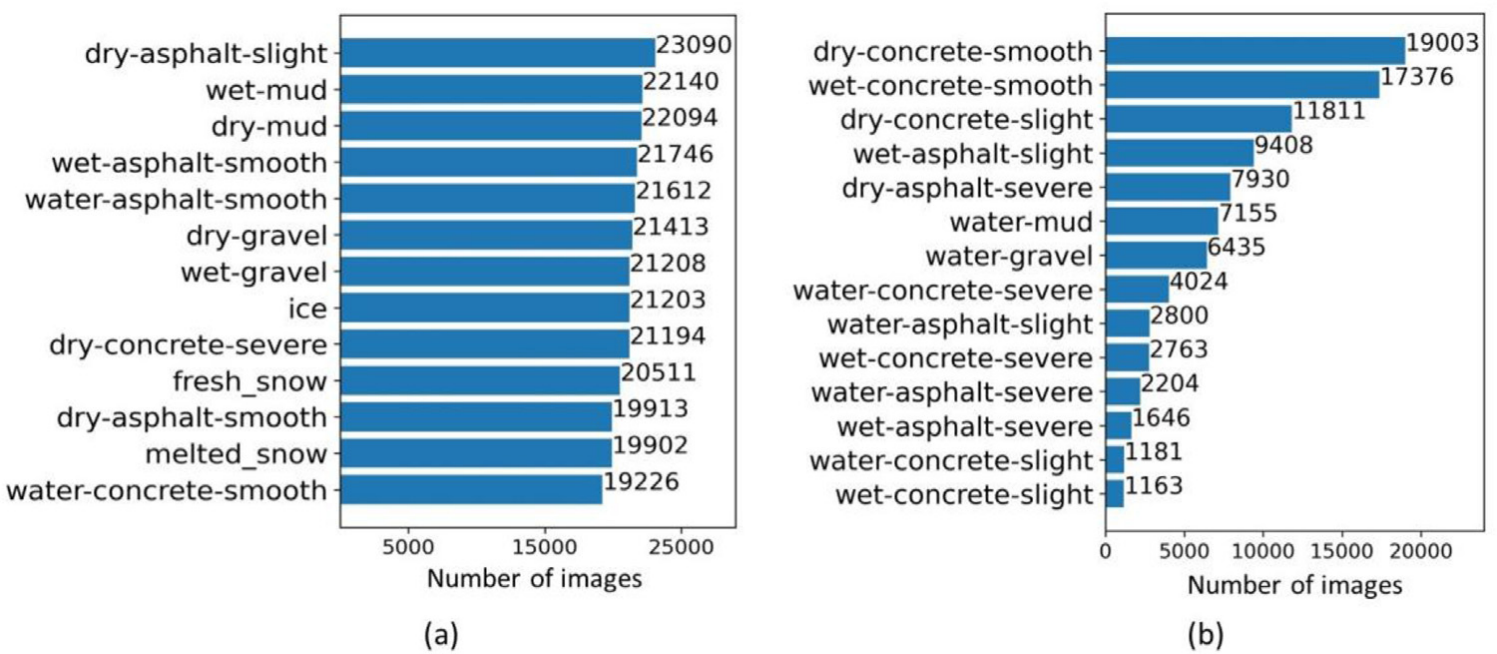
Number of images for each class. (a) number of the top 13 classes (b) number of the last 14 classes.

Based on the above classification strategies, 27 classes are defined. The directory structure of the dataset is shown in Fig. 1. There are 27 subfolders in the train-set folder, and each contains the images belonging to the class. All the 370151 training images (.jpg format) have the size of 240 × 360 pixels with a resolution of 96 dpi. Image samples of part of the classes are shown in Fig. 2. The number of images for each class is shown in Fig. 3.

To test the developed algorithms, 12 continuous image sequences with annotations are further provided in the test-set folder. Each of the sequence consists of 30 images with size 1920 × 1080, and 8 patches are labeled for every image. Taking the upper left corner of the image as the origin, the upper-left coordinates of the patches are (480, 200), (720, 200), (960, 200), (1200, 200), (480, 560), (720, 560), (960, 560), (1200, 560), respectively. The first coordinate value is along the width direction, while the height direction for the second. The labels for the patches of an image are stored as a .txt file in the same folder according to the coordinate order above.

## 2 Experimental Design, Materials and Methods

**Fig. 4:**
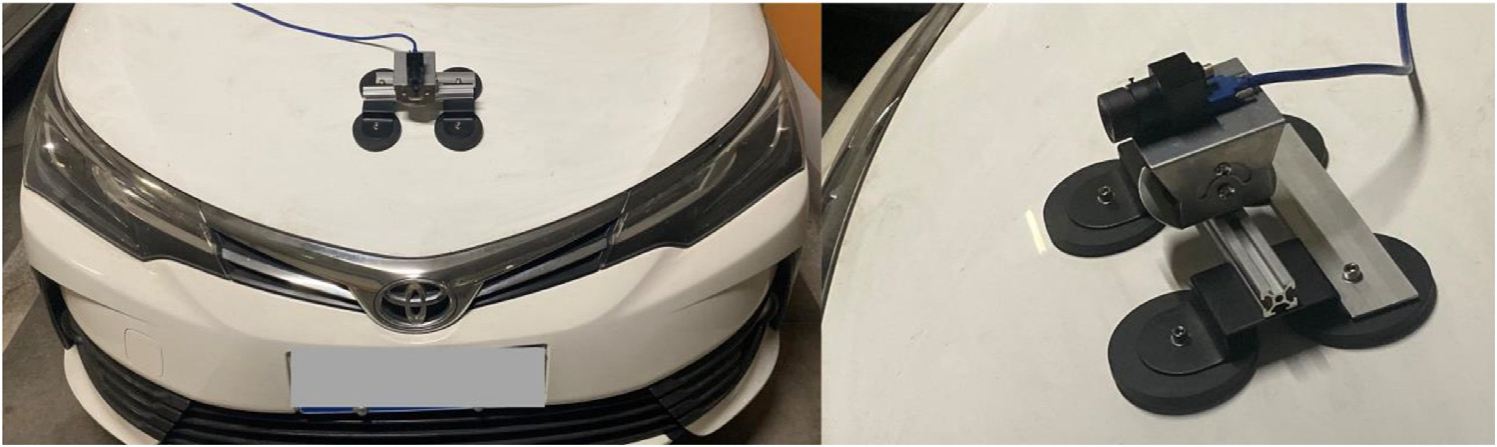
Camera installation.

The original pictures are acquired with a LI-USB30-AR023ZWDRB USB camera mounted on the vehicle bonnet, as shown in Fig. 4. Table 1 shows the specific parameters of the camera and the lens [6]. The camera has a certain depression angle to ensure the definition of the road surface area. The maximal preview distance on the flat road is 20 meters. The camera links to the IPC with a USB cable. The IPC runs a Python script that calls the OpenCV library to capture and store the pictures [7]. The system collects five pictures per second.

**Table 1:** Parameters of the camera and lens.

Camera		Lens	
Resolution	1920 × 1080	Aperture	1:1.4
ISP	AP0202	Focal length	6mm
Sensor size	1/2.7”	Mount	CS
Frame rate	30 fps	Distortion	0.35%
Max dynamic range	105 dB		

The pictures are captured from real roads accessible to the vehicle, which moves with a velocity in the range of 20-80km/h. The experiments are conducted in Beijing from October 2021 to May 2022 and cover various conditions of weather, sunlight brightness, road service age and aggregate characteristics, and also driving operation to enrich the pattern of the image dataset. Some corner cases such as debris on the road, dirt on the lens, and camera motion blur are also included. This dataset covers as many realistic situations as possible and lays a solid foundation for practical driving assistance applications.

Considering that the vehicle response is mainly affected by the area where the tire passes, we crop the original pictures into many patches with the size of 240 × 360 pixels for accurate road classification. This is realized with a Python script, after which the patches containing only the road surface areas are retained. Then the images are classified manually.

## Ethics Statement

This work did not include work involved with human subjects, animal experiments or data collected from social media platforms

## CRediT Author Statement

**Tong Zhao:** Methodology, Data curation, Software and Writing – original draft preparation; **Yintao Wei:** Supervision, Investigation, Project administration, Writing – review & editing.

## Acknowledgments

This research did not receive any specific grant from funding agencies in the public, commercial, or not-for-profit sectors.

## Declaration of Competing Interest

The authors declare that they have no known competing financial interests or personal relationships that could have appeared to influence the work reported in this paper.

## Data Availability

Road Surface Image Dataset with Detailed Annotations (Original data) (Mendeley Data).
